# Analysis of autoantibodies against Ro52/Ro60 in patients with suspected diagnosis of autoimmune systemic disease

**DOI:** 10.1186/1479-5876-10-S3-P41

**Published:** 2012-11-28

**Authors:** Iñaki Salvador, Estibaliz Ruiz-Ortiz, Alejandro Olivé, Domingo Escudero, Isabel Bielsa, Eva Martínez-Cáceres

**Affiliations:** 1 Dept. of Immunology (LIRAD-BST), Germans Trias Pujol University Hospital, UAB, Badalona, Spain; 2 Dept. of Rheumatology, Germans Trias Pujol University Hospital, UAB, Badalona, Spain; 3 Dept. of Neurology, Germans Trias Pujol University Hospital, UAB, Badalona, Spain; 4 Dept. of Dermatology, Germans Trias Pujol University Hospital, UAB, Badalona, Spain

## Background

Autoantibodies (Abs) to SSA/Ro recognize two ribonucleoproteins of 52- and 60-kDa. Ro52 antigen is an E3-ubiquitin-ligase. Anti-Ro52 Abs are present in patients with a broad spectrum of autoimmune diseases.

Usually these Abs are in association with anti-Ro60 in lupus erythematosus (SLE) and Sjögren syndrome (SS). However, isolated anti-Ro52 is frequently present in inflammatory myositis.

## Objective

To analyze the prevalence of anti-Ro52 and Ro60 Abs in a cohort of patients from a third level hospital with suspected diagnosis of autoimmunity.

## Materials and methods

Serum samples from 1100 patients, collected at the Immunology laboratory, HUGTP, from January 1999 until February 2009, that were tested for the presence of Abs against Extractable Nuclear Antigens (ENAs) using a commercial immunoblotting (Innolia) and ELISA assays (Orgentec). Most of the samples had a positive result in the anti-nuclear antibodies (ANAs) test.

Data were collected and analyzed in a retrospective way, and classified depending on the reported diagnostic in the medical history.

## Results

99 patients were Ro52+ Ro60- (group 1), 22 Ro52- Ro60+ (group 2), and 18 Ro52+ Ro60+ (group 3). In group 1, 7% patients had rheumatoid arthritis (RA) or other forms of autoimmune arthritis; 11% systemic sclerosis (SSc); 6% autoimmune hepatopathy (AH); 5% myositis; 20% SLE and 13% SS; Group 2 included 64% SLE, 4.5% SS, 4.5% psoriasis and 9% vasculitis. Finally, group 3 contained 39% SS, 28% SLE, 11% SSc, 5.5% AH, 5.5% RA.

## Conclusion

The finding of an isolated response to Ro52 is related mostly to SLE and SS, while an isolated response to Ro60 is present more frequently in SLE. In group 2 we found neither AR nor AH nor SSc. Markedly, patients diagnosed with myositis were only present in group 1.
